# Intranasal Lentiviral Vector-Mediated Antibody Delivery Confers Reduction of SARS-CoV-2 Infection in Elderly and Immunocompromised Mice

**DOI:** 10.3389/fimmu.2022.819058

**Published:** 2022-04-22

**Authors:** Yue Du, Shengnan Zhang, Zhaoyong Zhang, Kamran M. Miah, Peilan Wei, Lu Zhang, Yuhui Zhu, Zhengtu Li, Feng Ye, Deborah R. Gill, Stephen C. Hyde, Yanqun Wang, Jincun Zhao

**Affiliations:** ^1^Gene Medicine Research Group, Nuffield Department of Clinical Laboratory Sciences, Radcliffe Department of Medicine, University of Oxford, Oxford, United Kingdom; ^2^State Key Laboratory of Respiratory Disease, National Clinical Research Centre for Respiratory Disease, Guangzhou Institute of Respiratory Health, The First Affiliated Hospital of Guangzhou Medical University, Guangzhou, China; ^3^Health and Quarantine Laboratory, Guangzhou Customs District Technology Centre, Guangzhou, China; ^4^Institute of Infectious Disease, Guangzhou Eighth People’s Hospital of Guangzhou Medical University, Guangzhou, China; ^5^Guangzhou Laboratory, Guangzhou International Bio-Island, Guangzhou, China

**Keywords:** monoclonal neutralizing antibody, SIV.F/HN vectored immunoprophylaxis, COVID-19, old and immunodeficient mice, passive immunoprophylaxis

## Abstract

Vaccines for COVID-19 are now a crucial public health need, but the degree of protection provided by conventional vaccinations for individuals with compromised immune systems is unclear. The use of viral vectors to express neutralizing monoclonal antibodies (mAbs) in the lung is an alternative approach that does not wholly depend on individuals having intact immune systems and responses. Here, we identified an anti-severe acute respiratory syndrome coronavirus 2 (SARS-CoV-2) monoclonal antibody, NC0321, which can efficiently neutralize a range of SARS-CoV-2 variants, including alpha, beta, delta, and eta. Both prophylactic and therapeutic NC0321 treatments effectively protected mice from SARS-CoV-2 infection. Notably, we adopted viral vector-mediated delivery of NC0321 IgG1 as an attractive approach to prevent SARS-CoV-2 infection. The NC0321 IgG1 expression in the proximal airway, expressed by a single direct *in-vivo* intranasal (I.N.) administration of a self-inactivating and recombinant lentiviral vector (rSIV.F/HN-NC0321), can protect young, elderly, and immunocompromised mice against mouse-adapted SARS-CoV-2 surrogate challenge. Long-term monitoring indicated that rSIV.F/HN-NC0321 mediated robust IgG expression throughout the airway of young and SCID mice, importantly, no statistical difference in the NC0321 expression between young and SCID mice was observed. A single I.N. dose of rSIV.F/HN-NC0321 30 or 180 days prior to SARS-CoV-2 challenge significantly reduced lung SARS-CoV-2 titers in an Ad5-hACE2-transduced mouse model, reconfirming that this vectored immunoprophylaxis strategy could be useful, especially for those individuals who cannot gain effective immunity from existing vaccines, and could potentially prevent clinical sequelae.

## Introduction

Several coronavirus disease 2019 (COVID-19) vaccines have been approved for use globally (1); however, the protection provided by mRNA-based, viral vector-based, and conventional protein/virus subunit vaccines is modest in individuals with underlying conditions that can weaken the immune system. This is particularly pronounced in elderly adults where “booster” vaccine doses are being discussed to combat immunosenescence (2). Similarly, recent evidence suggests that only 60% of severely immunosuppressed patients with solid cancers can produce antibodies after the first dose of the Pfizer/BioNTech vaccine, while less than 15% of patients with blood or immune system cancers can produce antibodies (3). This is unsurprising, as previous data have suggested that age, immune status, and gender have a considerable effect on the immune response to flu vaccines (4). Therefore, there is an urgent need for alternative preventive measures independent of immune responses for more vulnerable populations—especially the elderly or those with compromised immune systems which are associated with more severe COVID-19.

Several monoclonal neutralizing antibodies (mNAbs) that can offer protection against severe acute respiratory syndrome coronavirus 2 (SARS-CoV-2) variants of concern (VOC) have been approved for clinical use (5). However, monoclonal antibody (mAb) therapy is costly and typically requires multiple doses to be clinically effective (6) and, thus, has not been used widely in the COVID-19 pandemic to date. Vectored immunoprophylaxis (VIP), based on neutralizing antibody gene transfer, has been proposed as an alternative strategy and is potentially beneficial to a diverse range of recipients regardless of immune status against a range of respiratory pathogens (7–9). A range of viral vectors have been proposed to mediate VIP, including a recombinant, self-inactivating, Simian immunodeficiency virus (SIV) vector pseudotyped with the fusion (F) and hemagglutinin–neuraminidase (HN) surface glycoproteins from Sendai virus (rSIV.F/HN). This vector mediates abundant, long-term, pulmonary transgene expression (7, 10–12). Crucially, the rSIV.F/HN vector offers potent transgene expression upon repeated administration, a feature not observed for at least some recombinant adeno-associated virus (rAAV) vectors also proposed for VIP (13). While rSIV./F/HN integrates its transgene expression cassette, this has not been associated with any integration site genotoxicity (10, 14). Together, these data suggest that rSIV.F/HN is a highly attractive gene transfer vector approach to deliver VIP for COVID-19 in a safe and effective manner.

We previously showed that the VIP strategy for COVID-19 (COVIP), in which NC0321 IgG1 (an ultrapotent anti-SARS-CoV-2 mAb) was expressed in the proximal airway after a single intranasal (I.N.) dose, conferred protection against a SARS-CoV-2 mimic in a mouse model expressing human angiotensin-converting enzyme 2 (hACE2) (15). Here, we extend our studies to compare the levels of protection observed in healthy young mice with healthy older mice as well as mice with severe immunodeficiency (NOD.Cg-PrkdcIL-2R-gamma null/SzJ, SCID). Also, we further investigate whether the immunoprophylaxis from a single I.N. dose of rSIV.F/HN.NC0321 could afford both short-term (30 days) and long-term (~6 months) prevention of authentic SARS-CoV-2 infection and replication in AdV5-hACE2-transduced mice. We believe that this rSIV.F/HN vector delivery of mAb by direct lung inhalation could be useful, especially for individuals who cannot gain effective immunity from existing vaccines, and could potentially prevent clinical sequelae for COVID-19 and other respiratory infections.

## Materials and Methods

### SARS-CoV-2 Virus

The SARS-CoV-2 strains used in this research were isolated from COVID-19 patients in Guangdong, China, including wild type (SARS-CoV-2/human/CHN/IQTC01/2020, NCBI, accession number: MT123290), alpha (B.1.1.7), beta (B.1.351), and eta (B.1.525). The SARS-CoV-2 delta (B.1.617.2) strain was provided by Guangdong Provincial Centre for Disease Control and Prevention, China. SARS-CoV-2 strains were passaged less than five times and titered using African Green monkey kidney-derived Vero E6 cells which were grown in Dulbecco’s modified Eagle’s medium (DMEM) supplemented with 10% fetal bovine serum (FBS). All work with SARS-CoV-2 was conducted in the Guangzhou Customs District Technology Center BSL-3 Laboratory.

### Isolation of Monoclonal Antibodies

Peripheral blood mononuclear cell (PBMC) isolation was performed *via* density gradient centrifugation over Ficoll-Paque, then IgG^+^ memory B cells were isolated from a cryopreserved COVID-19 patient’s PBMC by using CD22 microbeads (Miltenyi Biotec, Bergisch Gladbach, Germany) and immortalized with Epstein–Barr virus (EBV) as previously described (16). Culture supernatants were tested for their ability to bind SARS-CoV-2 proteins using enzyme-linked immunosorbent assay (ELISA). Positive cultures were collected and expanded. The VH and VL sequences from positive cultures were retrieved by reverse transcription-polymerase chain reaction (RT-PCR) and cloned into human IgG1 and Ig kappa or Ig lambda expression vectors as previously described (17). Monoclonal antibodies were produced by transient transfection of 293F cells (Invitrogen-Life technologies, Grand Island, USA). Supernatants from transfected cells were collected after 4 days, and IgG was affinity purified by protein A chromatography (GE Healthcare, Chicago, USA) and desalted against PBS.

### EC_50_ Determination by Enzyme-Linked Immunosorbent Assay

ELISA was used to determine the EC_50_ values of NC0321 against S, receptor-binding domain (RBD), S2, N-terminal domain (NTD), and C-terminal domain (CTD) proteins. Those proteins were coated onto 96-well plates (0.25 μg/ml) at 4°C overnight. Plates were blocked for 2 h with 10% FBS at 37°C. A serially diluted NC0321 antibody was added and incubated at 37°C for 2 h. After washing with PBST (0.1% Tween-20), HRP-conjugated mouse anti-human IgG (H+L) antibody (Jackson ImmunoResearch, West Grove, USA) as secondary antibody was added and incubated at 37°C for 1 h. TMB substrate solution was added and incubated for 10 min at RT, and the reaction was stopped by 2 M H_2_SO_4_. OD_450_ value was obtained using a microplate reader (BioTek Instruments, Inc.).

### Focus-Forming Assay for SARS-CoV-2 Quantification

All SARS-CoV-2 infection experiments were performed in a biosafety level 3 (BSL-3) laboratory. Concerning *in-vivo* challenge studies, mouse lungs were harvested and homogenized in PBS using a manual homogenizer. The virus was titered on Vero E6 cells. Vero E6 cells were seeded onto 96-well plates overnight and grown into confluent monolayers. Fifty microliters of 10-fold diluted SARS-CoV-2 stock or supernatant of lung homogenate was added into a 96-well plate and adsorbed at 37°C for 1 h with rocking every 10 min. The virus or supernatant of the lung homogenate was removed and covered with 100 μl MEM containing 1.2% carboxymethylcellulose (CMC). The plates were then incubated at 37°C for 24 h. Overlays were removed and cells were fixed with 4% paraformaldehyde solution for 2 h at room temperature. Cells were permeabilized with 0.2% Triton X-100 for 30 min at RT. Cells were then incubated with cross-reactive rabbit anti-SARS-CoV-N IgG (Cat: 40143-R001, Sino Biological, Inc., Beijing, China) as the primary antibody and HRP-conjugated goat anti-rabbit IgG (H+L) (Jackson ImmunoResearch, West Grove, USA) as the secondary antibody at 37°C for 1 h, respectively. The reactions were developed with KPL TrueBlue Peroxidase substrates. The numbers of SARS-CoV-2 foci were calculated using CTL ImmunoSpot S6 Ultra reader (Cellular Technology Ltd, Regions Great Lakes, Midwestern US.).

### Focus Reduction Neutralization Test of SARS-CoV-2

Focus reduction neutralization test (FRNT) assay was used for the evaluation of the NC0321 antibody neutralization effect. 1E5 Vero E6 cells were seeded into a 96-well plate 1 day before infection. Three-fold serially diluted NC0321 in DMEM was mixed with 100 FFU of SARS-CoV-2 (1:1). After 1 h incubation at 37°C, 50-μl mixtures were added into the 96-well plate seeded with Vero E6 cells, and the following steps were the same as the focus-forming assay (FFA) method described above. The IC_50_ is determined by 50% FRNT_50_ which was used for the evaluation of the potency of NC0321 in inhibiting SARS-CoV-2 replication.

### SARS-CoV-2 Surrogate Virus

The mouse-adapted (ma) SARS-CoV-2 surrogate, maS-LV (HIV1.SG614^Δ19aa^.CMV), based on a third-generation HIV1 vector, was pseudotyped with the SARS-CoV-2 spike protein Wuhan sequence (GenBank accession: 43740568) with three key mutations: D614G, Q498Y, and P499T (18, 19). Two maS-LV variants were used in this study: one directed the expression of firefly luciferase and the other enhanced green fluorescent protein (EGFP). The physical titer (ng p24) of maS-LV particles was determined using a p24 immunoassay (SEK11695, Sino Biological, Stratech, Cambridge, UK).

### Viral Vectors

For the Ad-hACE2 vector, the adenoviral vector expressing human angiotensin-converting enzyme 2 (hACE2) under the control of the CMV promoter was generated as previously described (20). For the rSIV.F/HN vector, the production and functional titration (transducing units/ml: TU/ml) of rSIV.F/HN vectors was performed as described previously (7, 10). The rSIV.F/HN viral vector genome plasmids included the hCEF transgene promoter, which is composed of cytosine guanine dinucleotide (CpG)-free CMV enhancer/elongation factor 1 alpha promoter (hCEF) (10), and the Woodchuck Hepatitis Virus Post-transcriptional Regulatory Element (WPRE) to enhance expression (21). Three mAb-containing rSV.F/HN vectors were used in this study, expressing NC0321, NC0321ΔAGG, and T1-3B, an anti-influenza IgG which was used as an isotype control (7); in each, the heavy and light chains of human IgG1 cDNAs were co-expressed in a single open reading frame (ORF) configuration (8). All mAb ORFs utilized two human growth hormone signal sequences for heavy and light chain secretion: an F2A self-cleaving peptide with a Furin cleavage site to separate the heavy and light chains and a mirT-142-3p 3′UTR sequence to improve immunologic tolerance (22). The production, purification, and titration of the recombinant adeno-associated virus rAAV2/9.hACE2 vector was conducted as previously demonstrated (15).

### Animal Studies

**Figure 1:**
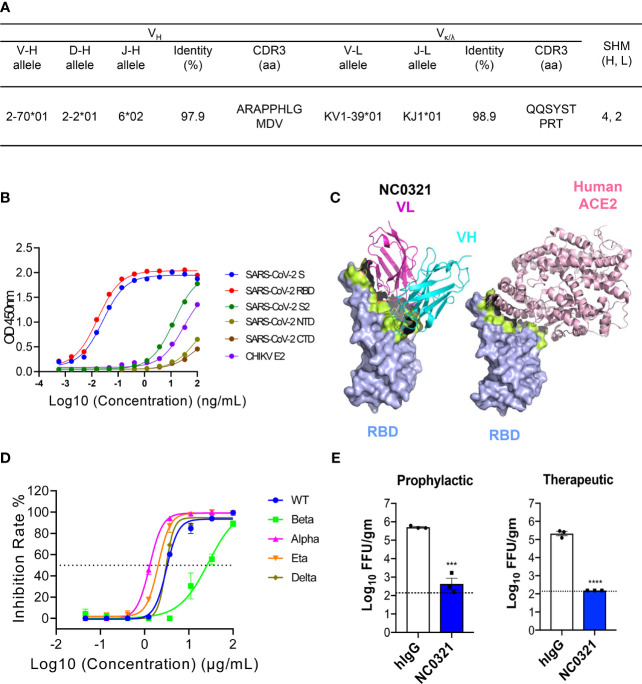
Isolation of the potent severe acute respiratory syndrome coronavirus 2 (SARS-CoV-2) human neutralizing antibody NC0321. **(A)** Germline analysis of the mAb NC0321. V-(D)-J rearrangement summary for the isolated mAb NC0321. **(B)** NC0321 isolated from a COVID-19 convalescent patient was tested for binding to SARS-CoV-2 spike (S), S1, S2, receptor-binding domain (RBD), N-terminal domain NTD, C-terminal domain CTD, and Chikungunya virus (CHIKV) E2 (as control) proteins in ELISA. **(C)** Computational models of SARS-CoV-2 RBD in complex with monoclonal antibody NC0312. Heavy (VH in cyan) and light (VL in purple) chains were colored as in **(B)**, and the structures of the complex between hACE2 and RBD were provided as a comparison. Residues that contribute substantially to interactions are colored in green. **(D)** Neutralization activity of mAb NC0321 against multiple live SARS-CoV-2 variants (wild type, alpha, beta, delta, and eta) in Vero E6 cells. **(E)** Passive transfer of NC0321 confers protection to mice prophylactically and therapeutically; we transferred 10 mg/kg NC0321 (200 µl in PBS) into Ad5-hACE2-transduced mice (6–8 weeks) intraperitoneally 1 day before or 1 day after the intranasal (I.N.) infection with 1 × 10^5^ FFU SARS-CoV-2 strain (SARS-CoV-2/human/CHN/IQTC01/2020, GenBank: MT123290.1). Virus titers in the lungs were measured at 3 dpi. **** indicate *p*-values of <0.0001, *** *p*-values of 0.0001<*p*<0.001.

For authentic virus challenge studies, we adopted the Ad5-hACE2-sensitized mice model, as previously described (20), to evaluate NC0321 performance *in vivo*. For the study shown in **Figure 1**, specific pathogen-free 5–6-week-old female BALB/c mice were anesthetized with isoflurane and transduced with 2.5E8 FFU of Ad5-hACE2 by I.N. delivery, and 5 days later, the mice were challenged with 1E5 FFU of SARS-CoV-2 (SARS-CoV-2/human/CHN/IQTC01/2020, NCBI, accession number: MT123290) by I.N. delivery. For prophylactic studies, mice were injected with 200 μg NC0321 by intraperitoneal (I.P.) delivery 1 day before SARS-CoV-2 infection. For therapeutic studies, mice were treated with NC0321 1 day after SARS-CoV-2 infection; human IgG was used as the negative control. For the short-term and long-term expression study shown in **Figure 4**, specific pathogen-free 5–6-week-old female BALB/c mice were I.N. dosed with 1E8 TU/ml rSIV.F/HN WT or ΔAAG vector for 30 or 180 days, respectively, and 2.5E8 FFU of AdV5-hACE2 administration (day −5) was intranasally delivered, followed by 1E5 FFU of authentic SARS-CoV-2 (wild-type strain) challenge *via* the I.N. route. In both studies, to determine the SARS-CoV-2 lung titer, mice were anesthetized on 1 or 3 days post-infection (dpi); lungs were removed into PBS and homogenized as previously described (20). Virus titers of clarified supernatants were assayed in Vero E6 cells and expressed as FFU/g of tissue.

**Figure 2:**
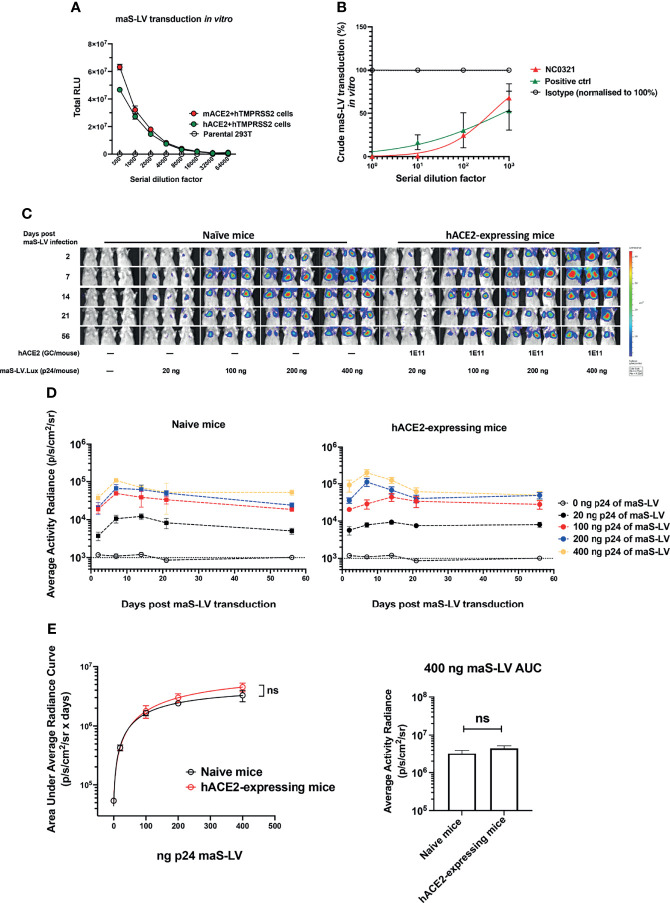
Generation of mouse-adapted SARS-CoV-2 surrogate permits lung transduction in wild-type mice. **(A)** Mouse-adapted SARS-CoV-2 surrogate (maS-LV) transduction in the parental HEK293T/17, mouse ACE2+TMPRSS2 or human ACE2+TMPRSS2 co-expressing HEK293T/17 cell line was determined (symbols represent mean±SEM, n=3). TMPRSS2, transmembrane protease serine 2 precursor, is important for SARS-CoV-2 spike priming as a serine protease (23). **(B)** Neutralization activity of NC0321 against an maS-LV expressing EGFP was confirmed by flow cytometry at the serial dilutions as indicated (n=3). An anti-spike antibody (40592-R001, Sino Biological) acted as positive control. **(C)** Bioluminescence imaging of mice (5-8 week old, female, n=3/group) at day 2, 7, 14, 21 and 56 dpi with the indicated p24 doses of a maS-LV expressing luciferase. Mice were naïve or received 1E11 Genome copies (GC) rAAV9.hACE2 14 days prior to maS-LV infection. **(D)** Time-course of bioluminescence imaging data for the indicated treatment groups after maS-LV infection at the indicated p24 dose ±hACE2 as indicated. The dotted line indicates the mean naïve background signal. **(E)** Area Under Curve (AUC) of bioluminescence (photons/sec/cm^2^/sr) values for each animal in **(C)** was computed, symbols represent mean±SEM for each treatment group (ANOVA, Dunnett’s multiple comparison of AUC between naïve and hACE2-expressing BALB/c mice). The right panel indicates the AUC in naïve and hACE2-expressing mice following treatment with 400ng p24 maS-LV (ns represents p > 0.05).

For studies including maS-LV titration and challenge, we adopted the rAAV2/9.hACE2-transduced mice model as previously described (15). For the study shown in **Figure 2**, two groups of BALB/c (5–8 weeks old, *n* = 3) were treated with PBS or 1E11 DRGC/ml (DNase-resistant genome copies/ml), respectively, *via* I.N. delivery. Fourteen days post-rAAV2/9.hACE2 vector delivery or mock treatment with PBS, mice were intranasally dosed with the titrating amount of maS-LV (20, 100, 200, and 400 ng p24). This was followed by bioluminescence imaging using an IVIS spectrum imaging system (IVIS Lumina LT, Series III, PerkinElmer) to determine *in-vivo* luciferase activity (photons/s/cm^2^/sr) values, as described previously (10). For the maS-LV challenge study in NC0321-treated protection study, young (5–8 weeks old) and old (7–8 months old) female and male BALB/c mice were purchased from Envigo RMS, UK. Severe immunodeficient NOD.Cg-Prkdc^IL-2R-gamma null^/SzJ SCID mice (female and male, 6 months old) were kind gifts from the MRC Human Immunology Unit (Dr. Uzi Gileadi, RDM, University of Oxford). Mice under light gaseous anesthesia received rSIV.F/HN vectors or maS-LV SARS-CoV-2 surrogates by I.N. delivery of a 100-µl volume *via* a single and continuous droplet. Mice were housed at the Biomedical Services Unit (BMS) (University of Oxford, John Radcliffe Hospital, Oxford, UK), and all animal procedures were performed with approval from the University of Oxford Animal Care and Safety.

### Human IgG Detection by ELISA

Mouse serum and bronchoalveolar lavage fluid (BALF) were collected as previously described (11). Human IgG expression in serum and BALF was measured using ELISA (Bethyl Laboratories, Cambridge Bioscience, Cambridge, UK). BALF human IgG levels were used to calculate lung epithelial lining fluid (ELF) human IgG levels *via* correction of the variable sample dilution achieved during BALF collection by correcting for BALF urea levels ab83362, Abcam, Cambridge, UK as previously described (24).

### Statistical Analysis

Group sizes were selected to achieve >0.8 power using G*Power 3.1.9.6 software (25). Statistical analysis was performed using GraphPad (San Diego, CA, USA) software. Where possible, one-way ANOVA followed by Dunnett’s multiple comparisons test was used to compare multiple treatment groups to a chosen comparator group or Tukey’s comparison of all groups. The non-parametric Kruskal–Wallis test with Dunnett’s multiple comparisons test was used to compare the experimental groups if the group number is less than 6 (*n* < 6). For bioluminescence studies, the area under the curve (AUC) of time-course studies was computed from individual animal data, and multiple comparisons of AUC between treatment groups were performed as described above. Errors were reported as the standard deviation of the median (SEM). A calculated *p*-value of <0.05 was considered as a significant difference. In figures, ns, *, **, ***, and **** indicate *p*-values of >0.05, <0.05, <0.01, <0.001, and <0.0001 respectively.

## Results

### Isolation of the Potent SARS-CoV-2 Human Neutralizing Antibody NC0321

To identify potent anti-SARS-CoV-2 human monoclonal antibodies, we isolated and combined serum and PBMCs at multiple points from one COVID-19 patient hospitalized in Guangzhou, China, during February 2020. The RT-PCR assay was utilized to confirm the presence of SARS-CoV-2 viral RNA in the patient’s nasopharyngeal swabs. IgG memory B cells were isolated from PBMCs by magnetic selection and immortalized by EBV transformation as previously described (16). We isolated single B cells after detecting SARS-CoV-2 spike (S) protein-specific antibodies in the culture supernatant. One mAb isolated from this approach, named NC0321, was purified and showed high binding activity with both SARS-CoV-2 S and RBD proteins by ELISA. Sequence analysis using IMGT databases (http://www.imgt.org/) indicated that NC0321 is derived from the VH2-70*01 and VK1-39*01 Ig gene repertoire, has an 11 amino acid (aa) heavy-chain complementarity determining region (HCDR) 3, and carries 4 aa substitutions in VH and 2 aa substitutions in VL (**Figure 1A**).

The NC0321 mAb bound SARS-CoV-2 S and RBD in a dose-dependent fashion with a half-maximal EC_50_ of 0.02596 and 0.01682 ng/ml (**Figure 1B**), respectively. Binding to three other S-related domains (SARS-CoV-2 NTD, CTD, and S2) and Chikungunya virus (CHIKV) E2 envelope protein was with substantially lower affinity than the S and RBD proteins (**Figure 1B**).

Molecular model building and computational docking was performed to predict the possible interactions between NC0321 and RBD (**Figure 1C**). The NC0321 footprint on the RBD partially overlaps with that of the human hACE2 receptor (**Supplementary Figure S1**), suggesting that NC0321-neutralizing activity results from interference with RBD–hACE2 interaction.

Subsequently, FRNT was used to evaluate the NC0321 mAb neutralization potential against authentic SARS-CoV-2. NC0321 displayed potent neutralization against SARS-CoV-2 wild type (WT, strain: SARS-CoV-2/human/CHN/IQTC01/2020, GenBank: MT123290.1), with an IC50 of 3.106 μg/mL(**Figure 1D**). We then sought to determine the cross-neutralizing reactivity against multiple SARS-CoV-2 VOC and variants of interest (VOI) (26); NC0321 showed neutralization activity against the SARS-CoV-2 alpha, beta, delta, and eta variants, with IC_50_ values of 1.3, 27.63, 3.014, and 2.041 μg/ml, respectively (**Figure 1D**). This broad neutralization activity against SARS-CoV-2 variants was consistent with the binding interactions suggested by the computational models. Importantly, these results suggest that NC0321, a mAb isolated from a recovering COVID-19 patient, has high neutralizing activity against the current repertoire of SARS-CoV-2 VOC and VOI circulating globally.

### The Prophylactic and Therapeutic Efficacy of NC0321 mAb Against SARS-CoV-2 in Ad5-hACE2-Transduced Mice

We sought to determine the prophylactic and therapeutic efficacy of NC0321 mAb in BALB/c mice sensitized to SARS-CoV-2 infection by prior transduction of a replication-deficient adenovirus (Ad5) engineered to express hACE2, the SARS-CoV-2 receptor (Ad5-hACE2) (20). Exogenous expression of hACE2 in murine lung cells enhances the cellular entry and, subsequently, the replication of live SARS-CoV-2. Ad5-hACE2-transduced mice were treated by I.P. injection of purified NC0321 mAb at 10 mg/kg either 1 day before (as the prophylactic group) or 1 day after (as the therapeutic group) the I.N. infection with 1E5 FFU SARS-CoV-2 (WT). In both prophylactic and therapeutic groups, NC0321 mAb treatment led to a 3-log reduction in lung viral titers at 3 dpi (**Figure 1E**). The data demonstrate that NC0321 mAb treatment can exert a profound prophylactic and therapeutic efficacy against SARS-CoV-2 *in vivo.*

### Generating Mouse-Adapted SARS-CoV-2 Surrogate Permits Lung Transduction in Wild-Type Mice

To simplify our *in-vivo* challenge studies, we sought to develop a murine SARS-CoV-2 infection model that did not require prior hACE2-humanization with Ad5-hACE2. A mouse-adapted, lentiviral vector-based, SARS-CoV-2 surrogate virus (maS-LV) was generated. This surrogate virus was able to utilize either hACE2 or murine ACE2 (mACE2) to efficiently infect cells *in vitro* (**Figure 2A**). Importantly, *in-vitro* cellular entry of maS-LV could be efficiently neutralized by NC0321 (**Figure 2B** and **Supplementary Figure S2A**).

We next sought to evaluate the performance of maS-LV *in vivo* and demonstrated that maS-LV could efficiently infect BALB/c mice independent of hACE2 (**Figure 2C**). Moreover, we observed a dose-dependent maS-LV-mediated luciferase expression in both naive and rAAV9-hACE2-pretreated BALB/c mice. Irrespective of maS-LV dose, luciferase expression peaked at approximately 7 dpi, fell modestly to a plateau at approximately 21 dpi, and was subsequently sustained for at least 56 dpi—consistent with lentiviral vector transduction kinetics (**Figure 2D**). Notably, luciferase expression resulting from maS-LV transduction in naive mice containing only endogenous mACE2 was comparable to that achieved in mice expressing hACE2 (ns, *p* > 0.05; **Figure 2E**, left panel). Importantly, both mice harboring mACE2 and hACE2 can facilitate similar luciferase expression when infected with a saturating dose of maS-LV (400 ng p24, **Figure 2E** right panel), suggesting that maS-LV infection is independent of hACE2 expression in mice. To simplify the experimental setting and avoid an oversaturating dose of the SARS-CoV-2 surrogate, WT BALB/c mice and 100 ng p24 of maS-LV were utilized in the subsequent challenge/protection studies.

### A Single Dose of rSIV.F/HN-NC0321 Protects Elderly and Immunocompromised Mice Against maS-LV Challenge

We next assessed whether NC0321 produced after *in-vivo* transduction with rSIV.F/HN-NC0321 vector can protect mice from maS-LV challenge. Additionally, we sought to evaluate any effects of mouse age (as a surrogate for immune system function) or host immune status. Thus, groups (*n* = 3) of young (5–8 weeks old) BALB/c and SCID mice were I.N. dosed with 5E8 TU rSIV.F/HN-NC0321. At 21 dpi, the expression of NC0321 IgG was assessed in BALF and ELF. Similar levels (*p* > 0.05) of NC0321 expression were noted in both groups (**Supplementary Figure S2B**).

**Figure 3:**
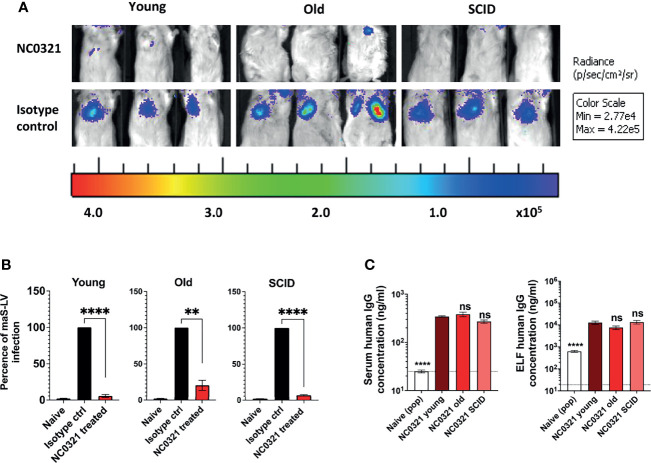
rSIV.F/HN mediated NC0321 mAb expression in young, old, and SCID mice against maS-LV infection. **(A)** Bioluminescence imaging of young (5-9 weeks weeks), old (7-8 months weeks), and SCID mice (6 months weeks) dosed with 5E8 TU rSIV.F/HN-NC0321 or an rSIV.F/HN vector expressing an irrelevant isotype control mAb; at 21 dpi, mice were challenged with 100 ng maS-LV-expressing luciferase. Imaging was performed 7 days post-maS-LV challenge. Representative images of *n* = 4–8/group are shown. **(B)** The bioluminescent value (photons/s/cm^2^/sr) for each animal in **(A)** was normalized such that the isotype control value was 100%. Naive values are from animals that did not receive maS-LV. Bars represent mean ± SEM (*t*-test; ** represents *p* = 0.0095 and **** represents *p* < 0.0001, *n* = 4–8 per group). **(C)** The human IgG levels in sera and epithelial lining fluid (ELF) in each group with NC0321 or isotype expressing vector transduction were determined. Human IgG levels in ELF were computed by comparison of urea levels in BALF and serum in every single sample (mean ± SEM, ordinary one-way ANOVA. ns represents *p* > 0.05 and **** represents *p* < 0.0001). Naive value, without urea normalization, is indicated by the dotted line.

Given that rSIV.F/HN mediated a similar IgG expression in the lungs of young and SCID mice, we next examined whether age and immune status affected the protective efficacy of rSIV.F/HN.NC0321 against challenge with maS-LV. Groups (*n* = 4–8) of young, old (7–8 months), and SCID mice were I.N. dosed with 5E8 TU rSIV.F/HN-NC0321 or an rSIV.F/HN vector expressing an irrelevant isotype control mAb (T1-3B) that does not neutralize SARS-CoV-2; at 21 dpi, mice were challenged with 100 ng p24 of a maS-LV-expressing luciferase. The weight of mice was monitored, and no obvious symptoms of distress were observed throughout the study (data not shown). On day 7 post-maS-LV challenge, bioluminescent luciferase activity in the lung was determined. High luciferase expression was noted in the lungs of mice treated with rSIV.F/HN expressing the isotype control compared with rSIV.F/HN.NC0321-dosed mice (**Figure 3A** and **Supplementary Figure S2C**). Vaccination with rSIV.F/HN-NC0321 conferred ~95.3%, 83.7%, and 93.1% protection of young, old, and SCID mice, respectively (**Figure 3B**).

At the end of the maS-LV challenge study, we evaluated NC0321 expression levels in both serum and ELF. All groups treated with rSIV.F/HN-NC0321 contained significantly more NC0321 mAb in the serum and ELF (**Figure 3C**) than wild-type animals (****, *p* < 0.0001). Young mice treated with rSIV.F/HN-NC0321 had comparable NC0321 mAb level in the serum (~3 µg/ml) and ELF (~10 µg/ml) to old and SCID mice (ns, *p* > 0.999). Moreover, comparable serum and ELF human IgG expression was observed in all groups despite differing gender balances (**Supplementary Figure S2D**).

### Short-Term and Long-Term Protection Efficacy of a Single Dose of rSIV.F/HN-NC0321 Against SARS-CoV-2 *In Vivo*

**Figure 4:**
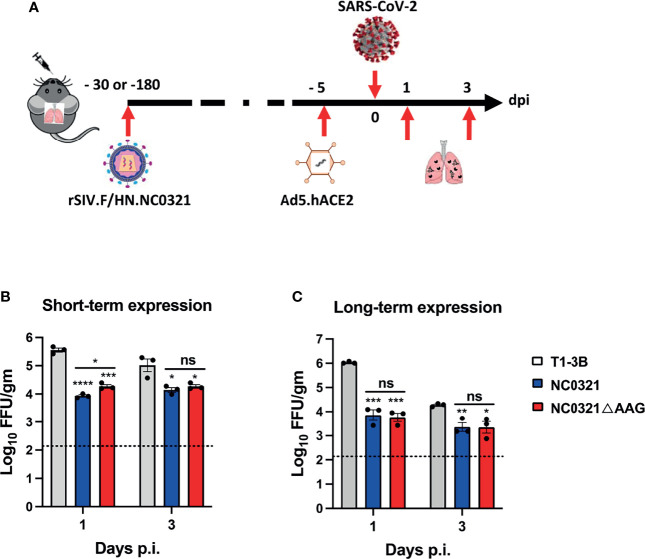
Protection against SARS-CoV-2 infection with vector-mediated NC0321 gene transfer. Viral load was measured in the lungs of groups (n = 3) of BALB/ c mice 1 and 3 dpi with the 1E5 FFU SARS-CoV-2 strain (SARS-CoV-2/human/CHN/IQTC01/2020, GenBank: MT123290.1). Groups had previously received 1E8 TU rSIV.F/HN-NC0321, rSIV.F/HN-NC0321DAAG, or rSIV.F/HN expressing an isotype control **(A)**, as indicated, 30 **(B)** or 180 days **(C)** previously. All animals received Ad5-hACE2 5 days prior to SARS-CoV-2 infection. Lungs were harvested for viral titers at 1 and 3 dpi (n = 3 mice per group). Dotted line indicates limit of detection (LOD); gm indicates gram lung tissue. ns, **, *** and **** represent p > 0.05, p < 0.05, < 0.001 and < 0.0001, respectively.

Having established that rSIV.F/HN-NC0321 could confer potent inhibition of maS-LV infection, we wished to evaluate the contribution of Fc effector functions. Thus, we introduced loss-of-function LALA-PG (L234A, L235A, and P329G), mutations into the Fc region of the NC0321 IgG1 heavy chain to generate rSIV.F/HN-NC0321ΔAGG. To explore the *in-vivo* immunoprophylaxis of rSIV.F/HN expressing NC0321 against wild-type SARS-CoV-2 infection, BALB/c mouse (*n* = 3/group) received a single I.N. administration of 1E8 TU of rSIV.F/HN-NC0321 or rSIV.F/HN-NC0321ΔAGG at 25 days (short-term protection group) or 175 days (long-term protection group) prior to 2.5E8 FFU I.N. Ad5-hACE2 transduction. Five days after Ad5-hACE2 transduction, mice were challenged with 1E5 PFU of SARS-CoV-2 WT. Lung tissues were harvested at days 1 and 3 post-SARS-CoV-2 infection for the analysis of viral load (experimental design, **Figure 4A**). In the short-term protection group, the rSIV.F/HN vector expressing NC0321 or NC0321ΔAGG both resulted in 1–1.5 log reduction in lung SARS-CoV-2 titers at day 1 and 1 log reduction on day 3 compared with the isotype control group (**Figure 4B**). In the long-term protection group, 180 days after rSIV.F/HN-NC0321 and rSIV.F/HN-NC0321ΔAGG vector delivery, both mAbs showed strong protection, with reduced viral loads of ~2 logs at day 1 and 1 log at day 3 (**Figure 4C**). Interestingly, no difference in protection efficacy was noted between rSIV.F/HN-NC0321 and rSIV.F/HN-NC0321ΔAGG treatment. Together, these data demonstrated that a single vaccination of rSIV.F/HN-NC0321 or rSIV.F/HN-NC0321ΔAGG can provide robust long-term IgG expression and afford remarkable long term (at least six months) prevention of SARS-CoV-2 replication in mouse lungs.

## Discussion

Despite a successful global vaccination program, the ongoing COVID-19 pandemic highlights an urgent unmet need for an effective immunization strategy for the millions of people around the globe who are clinically vulnerable with compromised immune systems (27). In this study, we demonstrated that a recombinant lentiviral vector, rSIV.F/HN, could be used to develop the VIP strategy against COVID-19. The approach does not require engagement of the hosts’ immune systems and was shown to be functional in both healthy and immunocompromised animal models of SARS-CoV-2 infection.

Several viral vectors have been investigated for the delivery of mAb genes to prevent respiratory infections in proof-of-principle studies (7, 9, 11, 28, 29). In particular, rSIV.F/HN has been successfully shown to be an effective platform for producing a stable, long-term (7), and prophylactic expression of mAbs (30) *in vivo* that provides protection against a range of infectious diseases, such as influenza (7) and RSV (11). In this study, we showed that the protection provided by rSIV.F/HN-NC0321 was similarly effective at both 30 and 180 days following I.N. administration, suggesting that the protection offered did not decrease over time; this is a major concern with both conventional vaccination (31) and adenoviral vector (32). Another benefit achieved with rSIV.F/HN vectors is the long-term transgene expression due, in part, to the integration of its transgene expression cassette within the host genome safe harbor (10, 14). Where required, the rSIV.F/HN vector can be engineered and developed to be integrase defective (IDLVs) to reduce the potential risk of insertional mutagenesis (33). Uniquely, rSIV.F/HN vectors can be effectively administered repeatedly (10, 12) allowing therapeutic antibody levels to be boosted if required and the addition of alternate ultrapotent single antibodies or antibody cocktails to counter newly emerging pathogenic variants and to counter immune escape. The key to an effective VIP strategy is an effective, potent mAb. From a recovering COVID-19 patient, we isolated NC0321, an ultrapotent broadly neutralizing human antibody that can neutralize a wide range of SARS-CoV-2 VOC *in vitro*. We anticipate that rSIV.F/HN-NC0321 can be readily translated to provide effective prophylaxis against SARS-CoV-2 VOC.

While NC0321 showed potent *in-vitro* neutralization of WT SARS-CoV-2 and the alpha, delta, and eta variants, we observed a reduction in neutralizing activity against the beta variant, suggesting that the beta variant may be able to partially escape VIP mediated by rSIV.F/HN-NC0321. Molecular modeling and computational docking suggested that this escape was potentially conferred by the combined mutations of the beta strain such as N501Y, E484K, and K417N. These key beta mutations could also favor the escape from conventional vaccine-induced immunity or immunity induced by natural infection with other variants (34). To limit the escape, NC0321 could be replaced or augmented by other ultrapotent mAbs (35, 36). Nevertheless, our assessment of the protection against SARS-CoV-2 VOC and VOC offered by NC0321 supports the use of VIP in those individuals who cannot gain effective immunity from existing vaccines.

In addition to evaluating NC0321, we also tested the VIP strategy mediated by a single I.N. delivery of rSIV.F/HN-NC0321ΔAAG, containing L234A, L235A, and P329G (LALA-PG) mutations in the Fc region of the NC0321 IgG1. NC0321ΔAAG may have a range of benefits, including disrupted mouse and human complement binding, reduced Fc-gamma/antibody-dependent and cell-mediated cytotoxicity, and enhanced *in-vivo* stability (37). Interestingly, in our limited studies, VIP delivery of NC0321 and NC0321ΔAAG resulted in similar anti-SARS-CoV-2 activity suggesting little or no advantage in our murine models of SARS-CoV-2 infection. Nevertheless, the NC0321ΔAAG variant may have advantages when evaluated in humans, and further evaluation in murine models may assist translation to human studies.

We used two murine models of SARS-CoV-2 infection, thanks to the exogenous human ACE2 expression expressed by rAAV2/9 or Ad5 vector. To minimize the impact of multiple viral vector administration events, we also used a SARS-CoV-2 surrogate that was able to utilize endogenous mACE2 to facilitate viral entry. This surrogate was generated by pseudotyping a lentiviral vector with the SARS-CoV-2 S protein. By introducing two key alterations (Q498Y and P499T) into the G614 variant of S as described previously (18, 19), we created maS-LV, a surrogate that efficiently utilized mACE2 (38). While native SARS-CoV-2 must be handled under difficult-to-access containment level 3 laboratories, one key advantage of the maS-LV surrogate was that it could be handled under simple standard laboratory conditions. This feature of maS-LV enabled a facile investigation of the efficacy of our VIP approach in elderly and immunodeficient animals. Immunocompromised mice, with B-, NK-, and T-cell deficiencies along with aberrant interferon signaling, are considered representative of humans with diminished immune systems (39). Crucially, our rSIV.F/HN-NC0321-based VIP approach appeared to be as effective in such immunocompromised animals as in animals with an intact immunological response.

Taken together, our results demonstrated that a single I.N. delivery of an rSIV.F/HN vector encoding NC0321 or NC0321ΔAAG, potent anti-SARS-CoV-2 mNAbs, can protect mice from authentic SARS-CoV-2 infection at or near the primary site of inoculation of the respiratory pathogen. This efficacy could be seen in mice regardless of their immune status, age, or gender. As anticipated by previous studies where rSIV.F/HN vectors direct life-long transgene expression in the lungs of research rodents, this protective effect was undiminished at 6 months post-vector delivery. We anticipate that our prophylactic approach could provide protection against respiratory diseases in all recipients; this includes, but is not limited to, vulnerable individuals who are unable to mount an effective immunological response to either SARS-CoV-2 infection or vaccination.

## Data Availability Statement

The original contributions presented in the study are included in the article/**Supplementary Material**. Further inquiries can be directed to the corresponding authors.

## Ethics Statement

The animal study was reviewed and approved, as appropriate, by Institutional Animal Care and Use Committees of the Guangzhou Medical University or the University of Oxford Committee on Animal Care and Ethical Review and by the UK Home Office. Written informed consent was obtained from the owners for the participation of their animals in this study.

## Author Contributions

YD designed and performed the maS-LV-related experiments and data analysis. KM designed and produced the maS-LV. SZ performed the authentic virus-related experiments and data analysis. ZZ, PW, LZ, YZ, ZL, and FY isolated and characterized the NC0321 sequence. DG, SH, and YW supervised the project and assisted in the experimental design. YD, SZ, and YW wrote the initial draft, with KM, DG, SH, and JZ providing editorial comments. All authors read and approved the manuscript.

## Funding

This project was supported by a Wellcome Trust Portfolio Grant Award No. 110579/Z/15/Z to the UK Respiratory Gene Therapy Consortium, Guangzhou Institute of Respiratory Health Open Project (funds provided by China Evergrande Group, 2020GIRHHMS07 and 2020GIRHHMS24), the National Natural Science Foundation of China (82172240, 82025001, 32000658, 81772191, 8217080414, 81861168032), Guangdong Province International Scientific and Technological Cooperation Projects (grant number 2020A0505100063), ZhongNanShan Medical Foundation of Guangdong Province (ZNSA-2020001), and State Key Laboratory of Respiratory Disease (SKLRD-Z-202214, SKLRD-Z-202007). For the purposes of open access, the authors have applied a CC BY public copyright license to any Author Accepted Manuscript version arising from this submission.

## Conflict of Interest

DG and SH hold IP in relation to rSIV.F/HN technology. Zhao holds IP in relation to NC0321.

The remaining authors declare that the research was conducted in the absence of any commercial or financial relationships that could be construed as a potential conflict of interest.

## Publisher’s Note

All claims expressed in this article are solely those of the authors and do not necessarily represent those of their affiliated organizations, or those of the publisher, the editors and the reviewers. Any product that may be evaluated in this article, or claim that may be made by its manufacturer, is not guaranteed or endorsed by the publisher.

## Supplementary Material

Supplementary Figure 1 Mapping of residues involved in the interaction between the RBD and NC0321. **(A)** Residues that contribute substantially to interactions between RBD and NC0321. **(B)** Prediction of crucial hydrogen-bonding residues for the interactions between RBD and NC0321.

Supplementary Figure 2 Prophylactic efficacy of rSIV.F/HN-NC0321 in mouse-adapted SARS-CoV-2 surrogate model. **(A)** The NC0321 IgG levels in BALF and ELF of groups of young and SCID mice were determined by ELISA 21 dpi with 5E8 TU rSIV.F/HN-NC0321.at the day of infection using ELISA assay (n = 3, t-test, ns represents p > 0.05). **(B)** Bioluminescence imaging value (photons/sec/cm2/sr) for each animal in **Figure 3A** was computed, symbols represent individual animals and bars represent group mean ± SEM (ANOVA, Dunnett’s multiple comparison between isotype and NC0321 treated BALB/c mice. ns represents p > 0.05, * represents p = 0.0381 and *** represents p < 0.001, n = 4-8 per group). **(C)** Bioluminescent imaging for each animal in **Figure 3A**. **(D)** Human IgG expression in sera (left) and ELF (light) of animals in **Figure 3A**, separated by gender (n = 3-4, ns represents p > 0.05, non-parametric analysis).
