# Supplementary material for: Intranasal Lentiviral Vector-Mediated Antibody Delivery Confers Reduction of SARS-CoV-2 Infection in Elderly and Immunocompromised Mice
Source: Front Immunol. 2022 Apr 22;13:819058. doi: 10.3389/fimmu.2022.819058 (PMC9072863; doi:10.3389/fimmu.2022.819058)
Supplement: Supplementary Figure 1 — Mapping of residues involved in the interaction between the RBD and NC0321. (A) Residues that contribute substantially to interactions between RBD and NC0321. (B) Prediction of crucial hydrogen-bonding residues for the interactions between RBD and NC0321. [file DataSheet_1.pdf]

Figure S1

A

RBD Site	ACE2	NC0321
403		R
405		D
406		E
408		R
409		Q
414		Q
415		T
416		G
417	K	K
421		Y
445	V	
446	G	G
449	Y	Y
453	Y	Y
455	L	L
456	F	F
473	Y	
475	A	A
476	G	G
477		S
484	E	E
485		G
486	F	F
487	N	N
488	Y	Y
493	Q	Q
494		S
495		Y
496	G	G
498	Q	Q
500	T	T
501	N	N
502	G	G
503	V	V
504		G
505	Y	Y

B

RBD	Length (Å)	NC0321
505Y	2.4	L96R
505Y	3.4	L91S
417K	2.4	L49Y
501N	2.2	L92Y
498Q	2.9	L92Y
498Q	2.9	L28S
494S	1.9	L30S
494S	3.3	L31S
494S	3.3	L31S
498Q	2.9	L28S

Figure S2

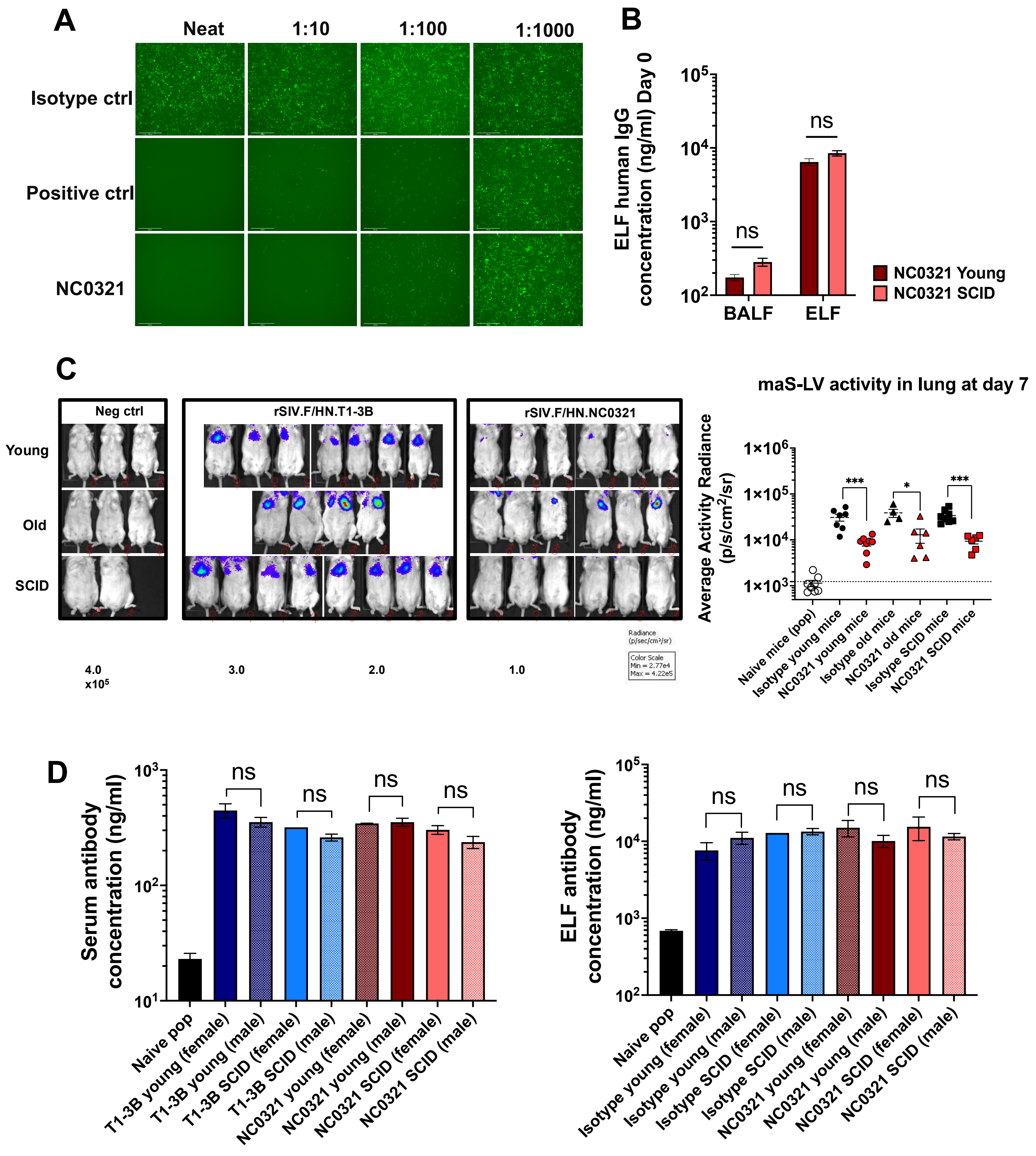

A
Neat
1:10
1:100
1:1000
Isotype ctrl
Positive ctrl
NC0321
B
ELF human IgG concentration (ng/ml) Day 0
ns
ns
BALF
ELF
NC0321 Young
NC0321 SCID
C
Neg ctrl
rSIV.F/HN.T1-3B
rSIV.F/HN.NC0321
Young
Old
SCID
4.0 x10⁵
3.0
2.0
1.0
Radiance (p/sec/cm²/sr)
Color Scale Min = 2.77e4 Max = 4.22e5
maS-LV activity in lung at day 7
Average Activity Radiance (p/s/cm²/sr)
***
*
***
Naive mice (pop)
Isotype young mice
NC0321 young mice
Isotype old mice
NC0321 old mice
Isotype SCID mice
NC0321 SCID mice
D
Serum antibody concentration (ng/ml)
ns
ns
ns
ns
Naive pop
T1-3B young (female)
T1-3B young (male)
T1-3B SCID (female)
T1-3B SCID (male)
NC0321 young (female)
NC0321 young (male)
NC0321 SCID (female)
NC0321 SCID (male)
ELF antibody concentration (ng/ml)
ns
ns
ns
ns
Naive pop
Isotype young (female)
Isotype young (male)
Isotype SCID (female)
Isotype SCID (male)
NC0321 young (female)
NC0321 young (male)
NC0321 SCID (female)
NC0321 SCID (male)
